# Baseline Glycated Hemoglobin Values Predict the Magnitude of Glycemic Improvement in Patients with Type 1 and Type 2 Diabetes: Subgroup Analyses from the DIAMOND Study Program

**DOI:** 10.1089/dia.2018.0163

**Published:** 2018-08-01

**Authors:** Liana K. Billings, Christopher G. Parkin, David Price

**Affiliations:** ^1^ Department of Clinical, NorthShore University HealthSystem, Skokie, Illinois.; ^2^ University of Chicago Pritzker School of Medicine, Chicago, Illinois.; ^3^ Department of Research, CGParkin Communications, Inc., Boulder City, Nevada.; ^4^ Department of Medical Affairs, Dexcom, Inc., San Diego, California.

**Keywords:** Type 1 diabetes, Type 2 diabetes, HbA_1c_, rtCGM, MDI, SMBG

## Abstract

The DIAMOND study demonstrated that the addition of real-time continuous glucose monitoring (rtCGM) effectively lowers glycated hemoglobin (HbA_1c_) in patients with type 1 (T1D) and type 2 diabetes (T2D), treated with multiple daily injections (MDI). This post hoc analysis investigated whether DIAMOND study participants at progressively higher baseline HbA_1c_ levels benefit from using rtCGM. We examined outcomes data from a large, randomized, controlled trial of MDI-treated participants with T1D (*N* = 158) and T2D (*N* = 158), comparing monitoring by rtCGM versus self-monitoring of blood glucose (SMBG). The primary outcome was the magnitude of HbA_1c_ reductions among study participants within elevated baseline HbA_1c_ levels (≥8.0%–10.0%, ≥8.5%–10.0%, and ≥9.0%–10.0%). Analyses were performed on three subgroups: T1D, T2D, and combined T1D/T2D. The full T1D analysis population had a mean baseline HbA_1c_ value of 8.6 ± 0.6% (range 7.5%–9.9%), randomized to rtCGM (*n* = 105) or control (*n* = 53). The full T2D analysis population had a mean baseline HbA_1c_ value of 8.5 ± 0.6% (range 7.5%–9.9%), randomized to rtCGM (*n* = 79) or control (*n* = 79). Participants had improvements in glycemic status regardless of monitoring method. In the three subgroups, the change in HbA_1c_ was significantly greater in rtCGM participants compared to SMBG at all predefined baseline HbA_1c_ levels at 12 and 24 weeks. Among the rtCGM participants, the change in HbA_1c_ was numerically greatest at the highest baseline HbA_1c_ subgroup (≥9.0%). Participants with elevated baseline HbA_1c_ had improvements in glycemic status regardless of monitoring method. However, the magnitudes of improvements appeared greater among participants using rtCGM.

## Previous Publications

The results reported in this study have not been previously reported. Primary results and other subanalyses have been previously published:

Beck RW, Riddlesworth T, Ruedy K, et al.: Effect of continuous glucose monitoring on glycemic control in adults with type 1 diabetes using insulin injections: the DIAMOND randomized clinical trial. JAMA 2017;317:371–378. Suppmenent 2: eTable 1 through eTable 10.
Ruedy KJ, Parkin CG, Riddlesworth TD, et al.: Continuous glucose monitoring in older adults with type 1 and type 2 diabetes using multiple daily injections of insulin: results from the DIAMOND trial. J Diabetes Sci Technol 2017;11:1138–1146.
Polonsky WH, Hessler D, Ruedy KJ, et al.: The impact of continuous glucose monitoring on markers of quality of life in adults with type 1 diabetes: further findings from the DIAMOND randomized clinical trial. Diabetes Care 2017;40:736–741.
Riddlesworth T, Price D, Cohen N, Beck RW: Hypoglycemic event frequency and the effect of continuous glucose monitoring in adults with type 1 diabetes using multiple daily insulin injections. Diabetes Ther 2017;8:947–951.
Beck RW, Riddlesworth TD, Ruedy KJ, et al.: Effect of initiating use of an insulin pump in adults with type 1 diabetes using multiple daily insulin injections and continuous glucose monitoring (DIAMOND): a multicenter, randomized controlled trial. Lancet Diabetes Endocrinol 2017;5:700–708.
Beck RW, Riddlesworth TD, Ruedy K, et al.: Continuous glucose monitoring versus usual care in patients with type 2 diabetes receiving multiple daily insulin injections: a randomized trial. Ann Intern Med 2017;167:365–374.

## Introduction

Studies have shown a greater magnitude of glycated hemoglobin (HbA_1c_) change at higher versus lower baseline HbA_1c_ levels following pharmacologic intervention in participants with type 1 (T1D) and type 2 diabetes (T2D).^1–3^ Meta-analyses have reported this effect in studies across 8 and 10 categories of noninsulin diabetes therapies, irrespective of medication class or mode of action.^4,5^ Furthermore, randomized, controlled trials in patients with T1D and T2D have demonstrated this effect as well when adding additional therapy such as dipeptidyl peptidase-4 inhibitors, sodium-glucose cotransporter-2 inhibitors, or glucagon-like peptide-1 mimetics to their insulin regimen.^1,6–11^ This phenomenon may be linked to the impact of chronic glucose toxicity on pancreatic ß-cell function,^12^ and that medications that address this condition would likely result in more significant HbA_1c_ reductions among individuals with extremely elevated baseline HbA_1c_ (characteristic of glucose toxicity) than in those with lower baseline levels when pancreatic ß-cell function is restored.

However, reducing glucose toxicity does not explain how interventions that modify behavior, such as real-time continuous glucose monitoring (rtCGM), would lower HbA_1c_ from higher levels. rtCGM measures glucose and provides users with glucose numbers, glucose trends, and alerts for impending or actual hypoglycemia and hyperglycemia. It remains uncertain if insulin-treated individuals with the worst glucose control—who may have contributing factors such as poor numeracy skills, poor medication or monitoring adherence, psychosocial issues, eating disorders, or profound fear of hypoglycemia—would have similar improvement in blood glucose control driven by change in behavior with the rtCGM data compared to people who are closer to HbA_1c_ goal.

In a 2011 meta-analysis, Pickup et al. observed that although rtCGM use was associated with a significant reduction in HbA_1c_ in patients with T1D, the largest reductions were seen in individuals with the highest HbA_1c_ values at baseline and in those who used their rtCGM device most frequently.^13^ The observation from this analysis has not yet been reported in a randomized clinical trial.

The recent DIAMOND study evaluated the effect of older generation rtCGM on glycemic control in multiple daily injection (MDI)-treated T1D and T2D adults with elevated HbA_1c_ levels. Results from analysis of the T1D and T2D study participants across a wide age range (26–79 years) showed that routine use of rtCGM compared with self-monitoring of blood glucose (SMBG) resulted in a greater decrease in HbA_1c_ level during 24 weeks and high rtCGM adherence.^14,15^ Similar benefits were observed across ages, educational levels, and numeracy skills of participants. A subsequent report from the T1D cohort found CGM to be cost-effective and a sensitivity analysis demonstrated even greater economic benefit with current CGM that eliminates the need for routine fingersticks and extends CGM wear duration.^16^

In this report, we present findings from a post hoc analysis that investigated the relationship between baseline HbA_1c_ thresholds and the magnitude of HbA_1c_ reductions among the DIAMOND study participants with elevated baseline levels (≥8.0%–10.0%). Additional analysis was conducted on the subgroup of participants with baseline HbA_1c_ ≥9.0%–10.0%, regarding their satisfaction with and adherence to rtCGM.

## Methods

The DIAMOND trial was composed of two independently powered trials in adult participants using multiple daily insulin injections, one with T1D and the second with T2D. The study was conducted at 27 endocrinology practices across North America. The study is listed on www.clinicaltrials.gov, under identifier NCT02282397. Details of the protocol and methods have been published^14,15^; relevant aspects of the protocol are summarized herein.

### Study participants

Major eligibility criteria for this analysis included age ≥25 years, diagnosis of T1D or T2D being treated with MDI of insulin for at least 1 year, central laboratory measured HbA_1c_ ≥7.5%–10.0%, stable diabetes medication regimen and weight over the prior 3 months, self-reported blood glucose meter testing averaging two or more times per day for T2D and three or more for T1D, and estimated glomerular filtration rate ≥45. Major exclusion criteria were use of rtCGM within 3 months of screening and any medical condition(s) that would make it inappropriate or unsafe to target an HbA_1c_ of <7.0% per investigator discretion.

### Study design

Details of the study design have previously been published.^14,15^ Participants in both groups received minimal, basic general diabetes education. Participants using rtCGM received limited device training and CGM management suggestions by a one-page tri-fold handout. This handout contained general guidelines about using rtCGM, and was reviewed at rtCGM initiation and week 4 and 12 visits. Clinicians provided individualized recommendations about each participant's goals and how to incorporate rtCGM trend information into their diabetes management. To have the study reflect clinical practice across the United States, specific insulin adjustments were not prescriptive in the protocol for either group, but instead were at the discretion of treating clinicians at the clinical sites. Follow-up clinic visits for both treatment groups occurred at 4, 12, and 24 weeks. There was only one scheduled study-related encounter before the final visit after week 4.

At week 24, satisfaction with rtCGM was assessed by completion of the CGM Satisfaction Survey (44 items on a 1–5 Likert scale, with the computed score representing the mean of the 44 items and subscales of benefits and lack of hassles).^17^ Adherence to CGM was assessed during the last month of the study. Self-reported insulin dosing frequency was reported at baseline and at week 24.

### Statistical methods

The primary outcome was change in the central laboratory-measured HbA_1c_ from baseline to 24 weeks; a secondary analysis measured HbA_1c_ change at 12 weeks. In this post hoc analysis, change in HbA_1c_ was stratified by baseline HbA_1c_ thresholds and comparisons between rtCGM and control groups. Treatment group comparisons were made with propensity scores,^18^ adjusted for baseline HbA_1c_ level and clinical site. For all analyses, missing HbA_1c_ values in which the central lab was missing, but local lab was known, were imputed using a regression line based on the site's local HbA_1c_ measurements (rtCGM/control: 1/0 at 12 weeks; 1/0 at 24 weeks). *P*-value <0.05 was considered significant to account for multiple comparisons. Analyses were conducted using SAS version 9.4 (SAS Institute, Cary, NC).

## Results

The DIAMOND studies included 158 T1D and 158 T2D participants treated with MDI; mean baseline values were 8.6% for both T1D study groups and 8.5% for both T2D study groups.^14,15^ The demographic characteristics of both analysis populations have been reported previously.^14,15^ This analysis included 131 T1D participants (rtCGM, *n* = 86; control, *n* = 45) and 120 T2D participants (rtCGM, *n* = 63; control, *n* = 57) with baseline HbA_1c_ ≥8.0%–10%, and excluded study participants with lower baseline HbA_1c_ values. Magnitude of HbA_1c_ change in the full cohort (baseline HbA_1c_ ≥7.5%–10%) is reported for comparison purposes.

**FIG. 1.**
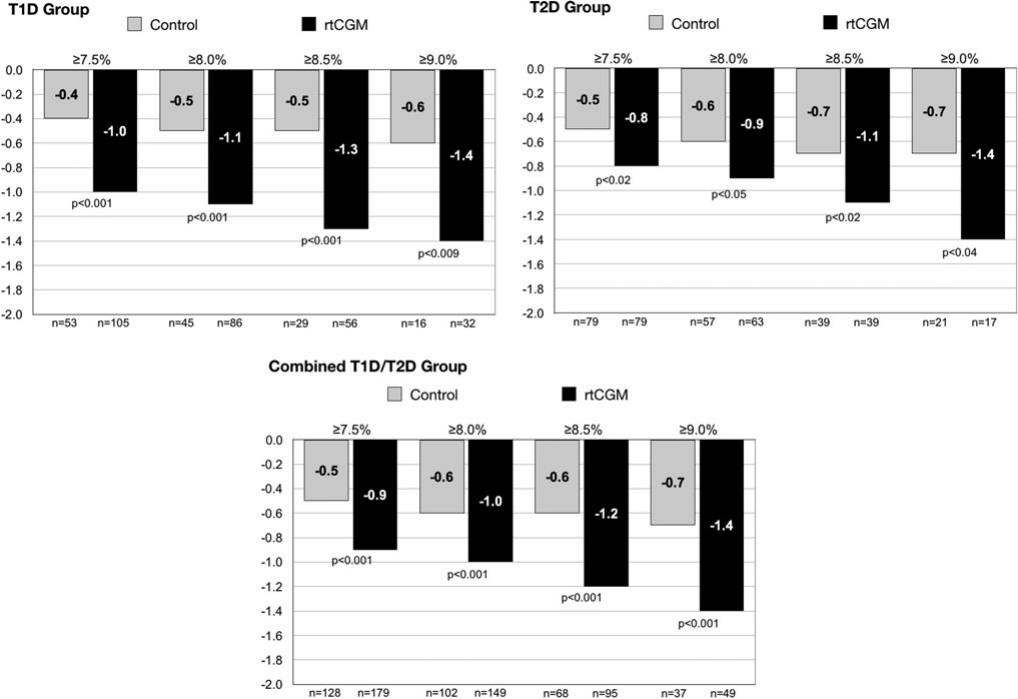
Comparisons of HbA_1c_ outcomes at 24 weeks according to baseline HbA_1c_ levels by patient subgroup. HbA_1c_, glycated hemoglobin; rtCGM, real-time continuous glucose monitoring; T1D, type 1 diabetes; T2D, type 2 diabetes.

In all study groups, the change in HbA_1c_ was significantly greater among participants in the rtCGM group compared to SMBG at all predefined HbA_1c_ thresholds at 12 and 24 weeks (Fig. 1). Among the rtCGM users, the change in HbA_1c_ was greatest in the highest HbA_1c_ subgroup (≥9.0%), with similar decreases seen in both the T1D and T2D groups. At 24 weeks, the impact of baseline HbA_1c_ on reductions was minimal in the T1D and T2D control groups.

Among participants with HbA_1c_ ≥9.0%, CGM satisfaction based on the CGM Satisfaction Survey demonstrated no difference versus those with a lower HbA_1c_ on perception of the benefits or lack of hassles with CGM. In addition, adherence remained high in those with HbA_1c_ ≥9.0% with 93% of the combined T1D and T2D CGM cohorts using CGM ≥6 days per week the last month of the study.

## Discussion

The DIAMOND study program demonstrated significant improvements in HbA_1c_ at 12 and 24 weeks in T1D and T2D participants who used rtCGM compared with those using a blood glucose meter alone for glucose monitoring.^14,15^ It is clear that use of rtCGM is an effective tool for patients with diabetes to improve control, but the question remained whether rtCGM use would benefit participants with the worst control in a low-touch clinical trial.

In this analysis, we observed a consistently greater reduction in HbA_1c_ at all baseline HbA_1c_ subgroups with rtCGM compared to SMBG. The greatest reductions were observed at the highest HbA_1c_ threshold ≥9%, corroborating the observations by Pickup et al.^13^ This pattern of greater reductions at higher baseline HbA_1c_ thresholds is not seen in the SMBG group and therefore cannot be attributed to enrollment in a clinical trial or regression to a mean, but rather the initiation of an effective behavioral intervention tool, rtCGM.

Unlike pharmaceutical studies, in which the investigators assess a given medication's known mechanism of action and glucose-lowering ability, medical device studies are more complicated. Understanding how behavior-based interventions function is more complex because the mode of action involves both device performance and behavioral response from study participants and/or their treating clinicians.

For example, in a survey of adults with T1D, Pettus et al.^19^ characterized diabetes management behaviors based on rtCGM that would translate into HbA_1c_ reduction. Survey respondents reported that rtCGM high-glucose alerts enabled them to respond to episodes of nocturnal hyperglycemia, which typically goes unrecognized and presents a potential large glycemic burden. The majority of survey respondents also stated that they took more insulin boluses or injections per day since starting on rtCGM. This was also observed in the SWITCH trial.^20,21^ In addition, most survey respondents reported adjusting their insulin timing relative to a meal and their meal insulin dose, based on the rtCGM trend arrows. Finally, many users lowered their glycemic targets since starting on CGM, which was likely related to less fear of hypoglycemia. Based on the results, it appears that the patients with poorly controlled diabetes made some of these diabetes management modifications based on their CGM data to derive significant benefit.

In this study, T1D and T2D participants with HbA_1c_ ≥9% randomized to rtCGM had a notable reduction in mean HbA_1c_ −1.4 ± 0.7 from baseline to study end. Compared to control group participants with HbA_1c_ >9.0%, this large reduction is similar to findings observed in trials that intensified therapy with additional medications in T1D and insulin-treated T2D with poorly controlled diabetes.^1,6–11^ Thus, we demonstrated that rtCGM has similar glycemic benefits compared to additional pharmacotherapy by empowering patients and clinicians, while eliminating the downsides of adding further medications.

Importantly, rtCGM use was sustained throughout this study,^14,15^ and participants noted high satisfaction with rtCGM in responses to the rtCGM Satisfaction Survey,^17^ with no difference in responses when examined by baseline HbA_1c_ levels. These findings support the hypothesis that high treatment satisfaction results in high adherence, and that high adherence results in glycemic benefit, even within the study population with the worst baseline control.

These findings also have implications for payers. Data from the NHANES survey demonstrated that 36.9% of insulin plus oral agent users and 49.1% of adult insulin-only users in the United States had an HbA_1c_ ≥8.0%.^22^ The landmark Diabetes Control and Complication Trial showed a curvilinear relationship between HbA_1c_ and risk of development and progression of complications—there was an exponential increase in risk observed as HbA_1c_ levels incrementally increased to higher levels.^23^ Patients with incrementally higher HbA_1c_ levels >7.5% have been shown to progressively increase health system costs,^24^ and have the greatest economic benefit from improving their glycemic control.^25^ A 1.0% reduction in HbA_1c_ from 10.0% to 9.0% is associated with $805 saving >3 years in adults with diabetes, but without heart disease and hypertension.^24^ The cost saving climbs to $1,130 in those with hypertension, $2,078 with heart disease, and $2,675 with both hypertension and heart disease.^24^ Among the 49 participants in the rtCGM group with a measured HbA_1c_ ≥9.0%, 20 had hypertension and 4 had diagnosed coronary disease.

## Conclusions

The objective of these analyses was to determine whether and to what degree high baseline HbA_1c_ values are associated with subsequent changes in glycemic status among MDI-treated T1D and T2D participants. As reported in this study, a positive relationship between high baseline HbA_1c_ values and improvements in glycemic status was observed among all study participants regardless of monitoring method. However, the magnitudes of improvements appeared greater among participants using rtCGM and were similar to those seen in pharmaceutical studies.^1–3,6–11^ Importantly, the improvements seen in patients with high baseline HbA_1c_ levels were achieved without the need for additional medications and associated costs. Thus, the costs of rtCGM in patients with high HbA_1c_ may be offset by avoiding treatment intensification with other medications and costs associated with medication side effects, and the longer-term savings achieved by lowering HbA_1c_ levels in poorly controlled diabetes populations.

## References

[1] Garg SK, Henry RR, Banks P, et al.: Effects of sotagliflozin added to insulin in patients with type 1 diabetes. N Engl J Med 2017;377:2337–2348 28899222 10.1056/NEJMoa1708337

[2] Giugliano D, Maiorino M, Bellastella G, et al.: Relationship of baseline HbA1c, HbA1c change and HbA1c target of <7% with insulin analogues in type 2 diabetes: a meta-analysis of randomised controlled trials. Int J Clin Pract 2011;65:602–612 21489084 10.1111/j.1742-1241.2010.02619.x

[3] Caputo S, Andersen H, Kaiser M, et al.: Effect of baseline glycosylated hemoglobin A1c on glycemic control and diabetes management following initiation of once-daily insulin detemir in real-life clinical practice. Endocr Pract 2013;19:462–470 23337147 10.4158/EP12269.OR

[4] Esposito K, Chiodini P, Bellastella G, et al.: Proportion of patients at HbA1c target <7% with eight classes of antidiabetic drugs in type 2 diabetes: systematic review of 218 randomized controlled trials with 78 945 patients. Diabetes Obes Metab 2012;14:228–233 21958121 10.1111/j.1463-1326.2011.01512.x

[5] DeFronzo RA, Stonehouse AH, Han J, Wintle ME: Relationship of baseline HbA1c and efficacy of current glucose-lowering therapies: a meta-analysis of randomized clinical trials. Diabet Med 2010;27:309–317 20536494 10.1111/j.1464-5491.2010.02941.x

[6] Yang W, Cai X, Gao X, et al.: Addition of dipeptidyl peptidase-4 inhibitors to insulin treatment in type 2 diabetes patients: a meta-analysis. J Diabetes Investig 2018;9:813–821 10.1111/jdi.12764 PMC6031492 29047219

[7] Inagaki N, Harashima S, Maruyama N, et al.: Efficacy and safety of canagliflozin in combination with insulin: a double-blind, randomized, placebo-controlled study in Japanese patients with type 2 diabetes mellitus. Cardiovasc Diabetol 2016;15:89 27316668 10.1186/s12933-016-0407-4 PMC4912792

[8] Vilsbøll T J Rosenstock J, Yki-Järvinen H, et al.: Efficacy and safety of sitagliptin when added to insulin therapy in patients with type 2 diabetes. Diabetes Obes Metab 2010;12:167–177 20092585 10.1111/j.1463-1326.2009.01173.x

[9] Terauchi Y, Tamura M, Senda M, et al.: Long-term safety and efficacy of tofogliflozin as add-on to insulin in patients with type 2 diabetes: results from a 52-week, multicentre, randomized, double-blind, open-label extension, Phase 4 study in Japan (J-STEP/INS). Diabetes Obes Metab 2018;20:1176–1185 29316236 10.1111/dom.13213 PMC5947124

[10] Min SH, Yoon J-H, Hahn S, Cho YM: Comparison between SGLT2 inhibitors and DPP4 inhibitors added to insulin therapy in type 2 diabetes: a systematic review with indirect comparison meta-analysis GLP-1 mimetics. Diabetes Metab Res Rev 2017;33:e2818 10.1002/dmrr.2818 27155214

[11] Yki-Järvinen H, Rosenstock J, Duran-Garcia S: Effects of adding linagliptin to basal insulin regimen for inadequately controlled type 2 diabetes: a ≥52-week randomized, double-blind study. Diabetes Care 2013;36:3875–3881 24062327 10.2337/dc12-2718 PMC3836100

[12] Campos C: Chronic hyperglycemia and glucose toxicity: pathology and clinical sequelae. Postgrad Med 2012;124:90–97 23322142 10.3810/pgm.2012.11.2615

[13] Pickup JC, Freeman SC, Sutton AJ: Glycaemic control in type 1 diabetes during real time continuous glucose monitoring compared with self monitoring of blood glucose: meta-analysis of randomised controlled trials using individual patient data. BMJ 2011;343:d3805 21737469 10.1136/bmj.d3805 PMC3131116

[14] Beck RW, Riddlesworth T, Ruedy K, et al.: Effect of continuous glucose monitoring on glycemic control in adults with type 1 diabetes using insulin injections: the DIAMOND randomized clinical trial. JAMA 2017;317:371–378 28118453 10.1001/jama.2016.19975

[15] Beck RW, Riddlesworth TD, Ruedy K, et al.: Continuous glucose monitoring versus usual care in patients with type 2 diabetes receiving multiple daily insulin injections: a randomized trial. Ann Intern Med 2017;167:365–374 28828487 10.7326/M16-2855

[16] Huang ES, O'Grady M, Basu A, et al.: The cost-effectiveness of continuous glucose monitoring in type 1 diabetes. Diabetes Care 2010;33:1269–1274 20332354 10.2337/dc09-2042 PMC2875436

[17] Juvenile Diabetes Research Foundation Continuous Glucose Monitoring Study Group: Validation of measures of satisfaction with and impact of continuous and conventional glucose monitoring. Diabetes Technol Ther 2010;12:679–684 20799388 10.1089/dia.2010.0015 PMC3045572

[18] Rosenbaum PR, Rubin DB: Reducing bias in observational studies using subclassification on the propensity score. J Am Stat Assoc 1984;79:516–524

[19] Pettus J, Price DA, Edelman SV: How patients with type 1 diabetes translate continuous glucose monitoring data into diabetes management decisions. Endocr Pract 2015;21:613–620 25716635 10.4158/EP14520.OR

[20] Wysham C, Bhargava A, Chaykin L, et al.: Effect of insulin degludec vs insulin glargine U100 on hypoglycemia in patients with type 2 diabetes: the SWITCH 2 randomized clinical trial. JAMA 2017;318:45–56 28672317 10.1001/jama.2017.7117 PMC5817473

[21] Lane W, Bailey TS, Gerety G, et al.: Effect of insulin degludec vs insulin glargine U100 on hypoglycemia in patients with type 1 diabetes: the SWITCH 1 randomized clinical trial. JAMA 2017;318:33–44 28672316 10.1001/jama.2017.7115 PMC5817477

[22] Selvin E, Parrinello CM, Daya N, Bergenstal RM: Trends in insulin use and diabetes control in the U.S.: 1988–1994 and 1999–2012. Diabetes Care 2016;39:e33–e35 26721815 10.2337/dc15-2229 PMC4764038

[23] The Diabetes Control and Complications Trial Research Group: The effect of intensive treatment of diabetes on the development and progression of long-term complications in insulin-dependent diabetes mellitus. N Engl J Med 1993;329:977–986 8366922 10.1056/NEJM199309303291401

[24] Gilmer TP, O'Connor PJ, Rush WA, et al.: Predictors of health care costs in adults with diabetes. Diabetes Care 2005;28:59–64 15616234 10.2337/diacare.28.1.59

[25] Baxter M, Hudson R, Mahon J, et al.: Estimating the impact of better management of glycaemic control in adults with type 1 and type 2 diabetes on the number of clinical complications and the associated financial benefit. Diabet Med 2016;33:1575–1581 26773733 10.1111/dme.13062

